# Supplementary material for: Exploitation of ATP-sensitive potassium ion (KATP) channels by HPV promotes cervical cancer cell proliferation by contributing to MAPK/AP-1 signalling
Source: Oncogene. 2023 Jul 13;42(34):2558–77. doi: 10.1038/s41388-023-02772-w (PMC10439009; doi:10.1038/s41388-023-02772-w)
Supplement: Supplementary file 1 — Supplementary material [file 41388_2023_2772_MOESM1_ESM.pdf]

Supplementary Figure Legends

S1 Fig. K_{ATP} channels are important for HPV gene expression in cervical cancer cells.

A) Expression levels of *E6* and *E7* mRNA in HeLa cells treated with tolbutamide (200 µM) measured by RT-qPCR. Samples were normalised against *U6* mRNA levels. **B)** Representative western blots of E6 and E7 expression in HeLa cells treated with increasing doses of tolbutamide. GAPDH served as a loading control. **C)** Mean $DiBAC_4(3)$ fluorescence levels in HeLa cells treated with increasing dose of tolbutamide. Samples were normalised to DMSO control. Data represent means ± SD of three biological replicates. *$P<0.05$, **$P<0.01$, ***$P<0.001$, ****$P<0.0001$ (Student's t-test).

S2 Fig. Depletion of SUR2 has no impact on HPV gene expression or proliferation in cervical cancer cells.

A) Relative expression of *ABCC9B* mRNA in HeLa and SiHa cells transfected with a pool of SUR2-specific siRNA measured by RT-qPCR. Samples were normalised against *U6* mRNA levels. **B)** Relative mean $DiBAC_4(3)$ fluorescence levels in HeLa and SiHa cells transfected with SUR2 siRNA. **C)** Relative expression of *E6* and *E7* mRNA in HeLa and SiHa cells transfected with SUR2 siRNA measured by RT-qPCR. Samples were normalised against *U6* mRNA levels. **D)** Representative western blots of E6 and E7 expression in HeLa and SiHa cells transfected with SUR2 siRNA. GAPDH served as a loading control. **E-G)** Growth curve analysis **(E)**, colony formation assay **(F)** and soft agar assay **(G)** of HeLa and SiHa cells after transfection of SUR2-specific siRNA. Data represent means ± SD of three biological replicates with individual data points displayed. *Ns* not significant, *$P<0.05$, **$P<0.01$, ***$P<0.001$ (Student's t-test).

S3 Fig. Depletion of Kir6.2 reduces HPV gene expression and proliferation in cervical cancer cells.

A) Relative expression of *KCNJ11* mRNA in HeLa and SiHa cells transfected with a pool of Kir6.2-specific siRNA measured by RT-qPCR. Samples were normalised against *U6* mRNA levels. **B)** Relative mean DiBAC$_4$(3) fluorescence levels in HeLa and SiHa cells transfected with Kir6.2 siRNA. **C)** Relative expression of *E6* and *E7* mRNA in HeLa and SiHa cells transfected with Kir6.2 siRNA measured by RT-qPCR. Samples were normalised against *U6* mRNA levels. **D)** Representative western blots of E6 and E7 expression in HeLa and SiHa cells transfected with Kir6.2 siRNA. GAPDH served as a loading control. **E-G)** Growth curve analysis **(E)**, colony formation assay **(F)** and soft agar assay **(G)** of HeLa and SiHa cells after transfection of Kir6.2-specific siRNA. Data shown is means ± SD of three biological replicates with individual data points displayed where appropriate. *P<0.05, **P<0.01, ***P<0.001, ****P<0.0001 (Student's t-test).

S4 Fig. Stable suppression of SUR1 expression decreases the proliferation of cervical cancer cells.

Growth curve analysis **(A)**, colony formation assay **(B)** and soft agar assay **(C)** of monoclonal HeLa cell lines stably expressing either a non-targeting (shNTC) or a SUR1-specific shRNA. Data shown is means ± SD of three biological replicates with individual data points displayed where appropriate. *P<0.05, **P<0.01, ***P<0.001, ****P<0.0001 (Student's t-test).

S5 Fig. K_{ATP} channel inhibition does not impact upon the survival of cervical cancer cells.

A) Representative western blots of PARP and caspase 3 cleavage in HeLa and SiHa cells treated with DMSO or glibenclamide (10 μM) for the indicated durations. Staurosporine treatment (STS, 1 μM for 6 hours) served as a positive control for apoptosis induction. GAPDH served as a loading control. **B)** Flow cytometry analysis of Annexin V assay using HeLa and

SiHa cells treated with DMSO or glibenclamide (10 µM) for the indicated durations. Bars represent means ± SD of three biological replicates. *Ns* not significant (Student's t-test).

S1 Table. List of primers used for RT-qPCR in this study.

Supplementary Figure 1

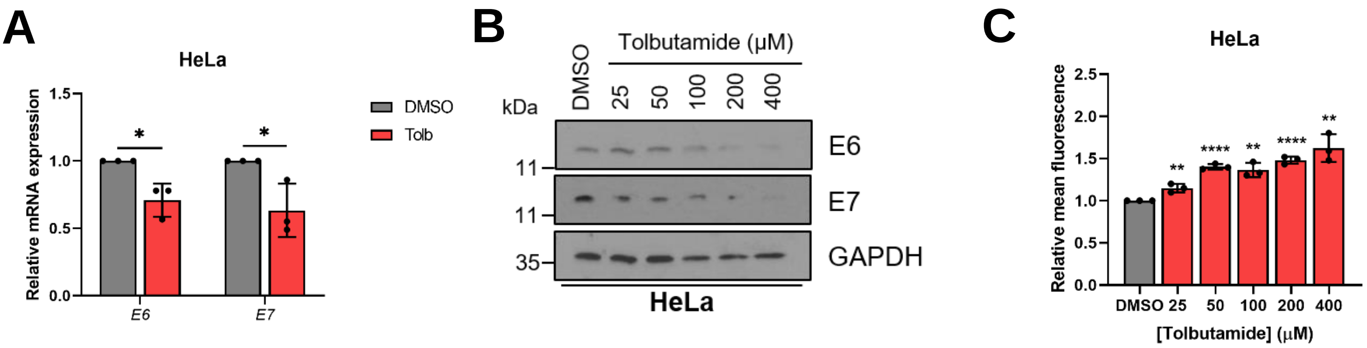

Supplementary Figure 2

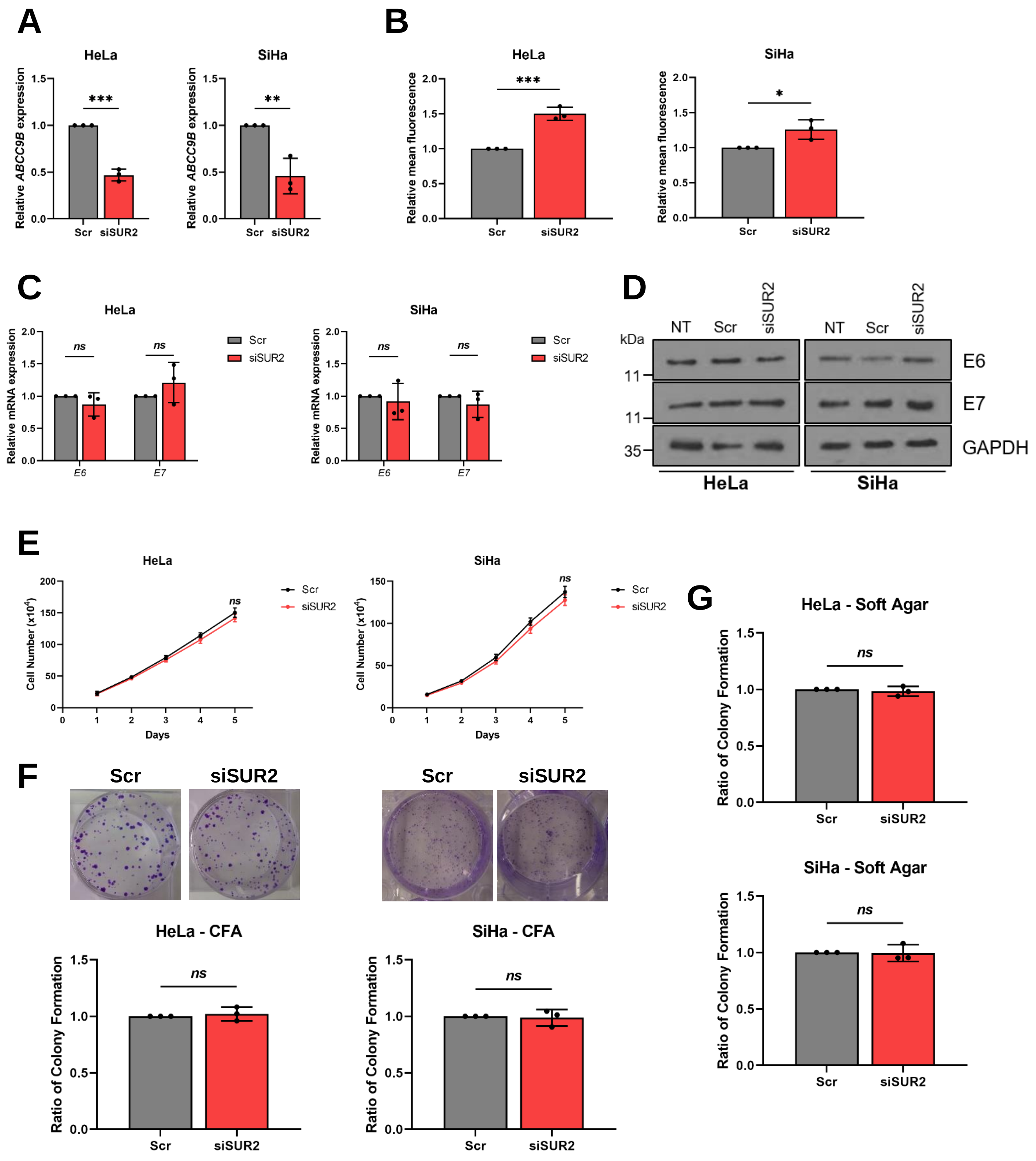

Supplementary Figure 3

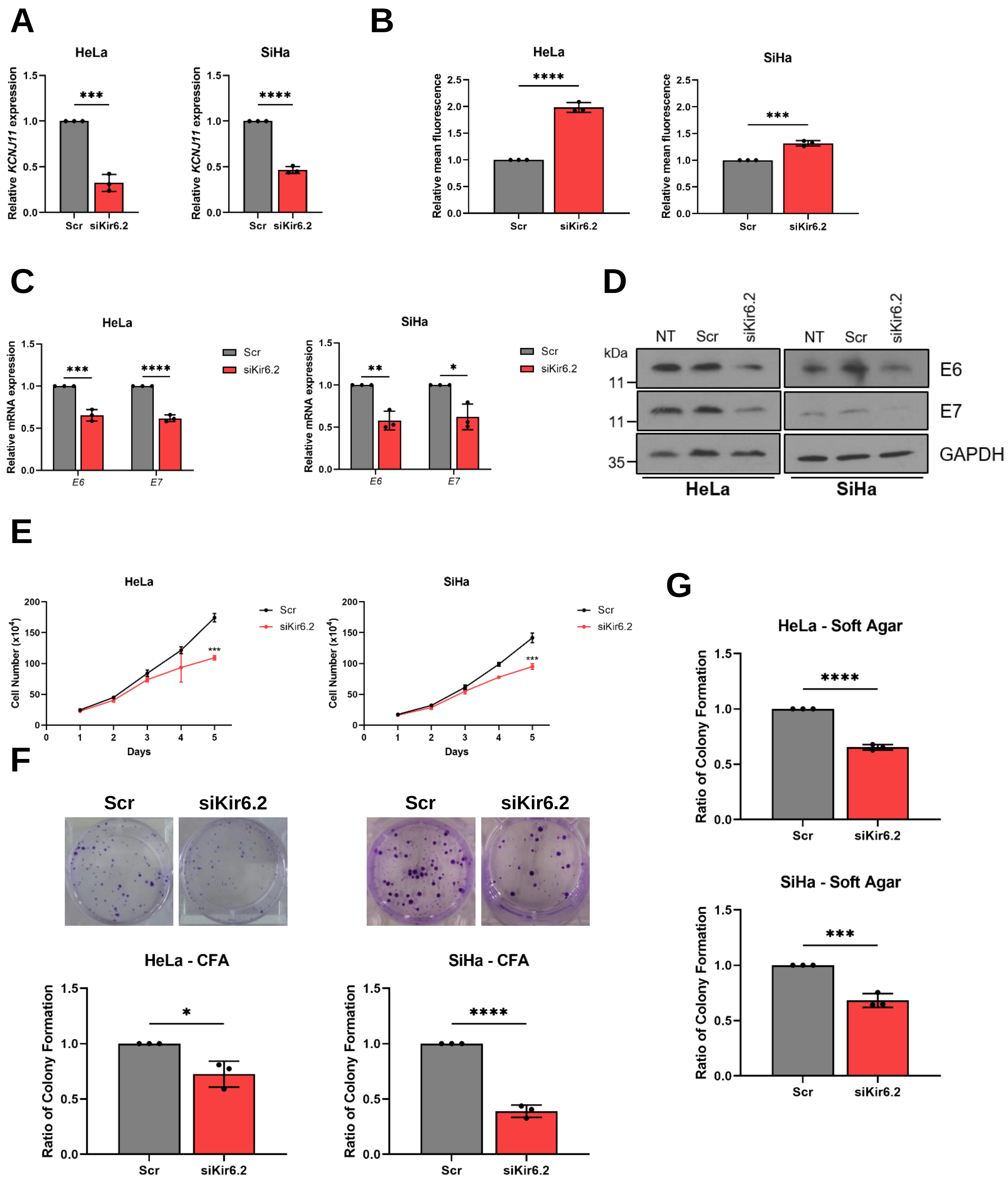

Supplementary Figure 4

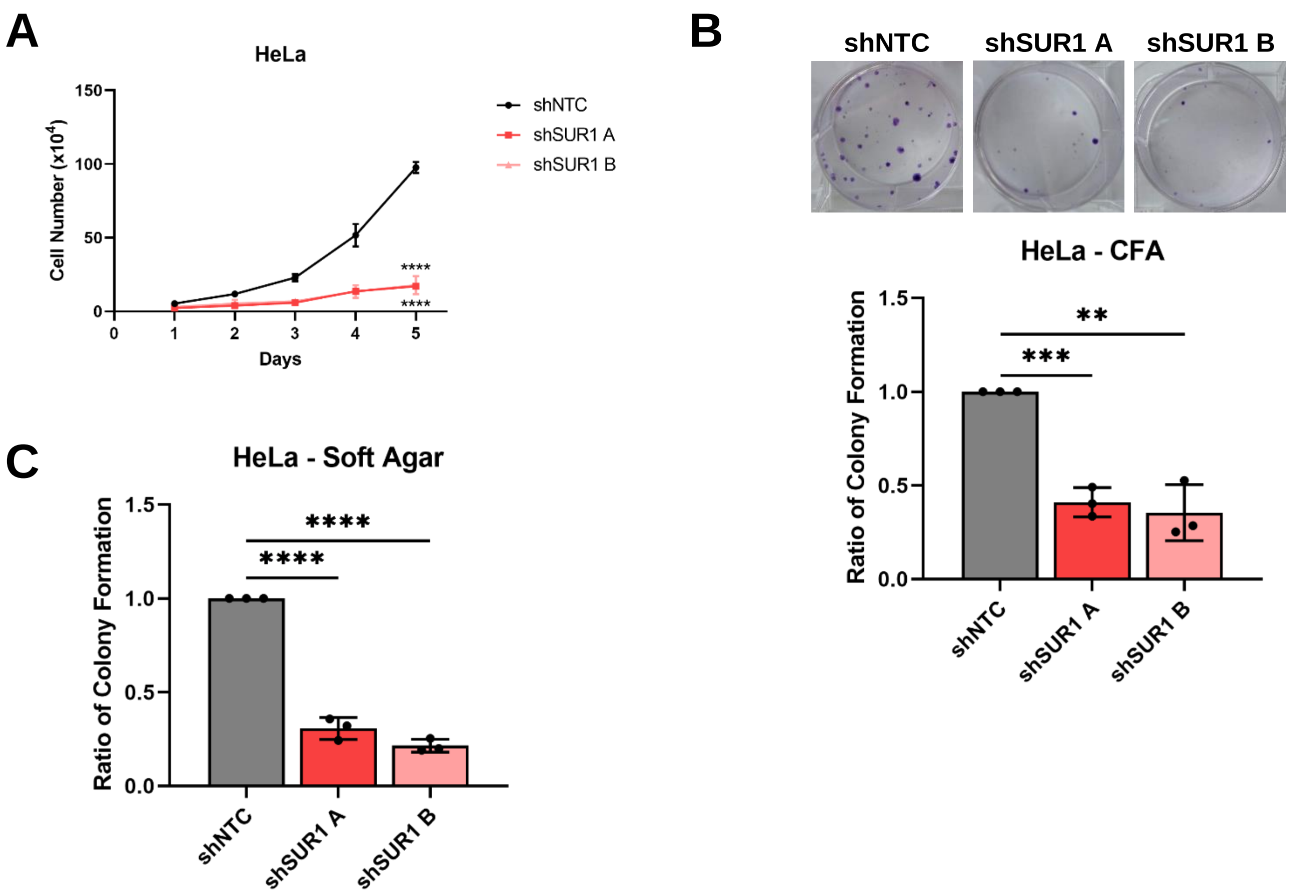

Supplementary Figure 5

A

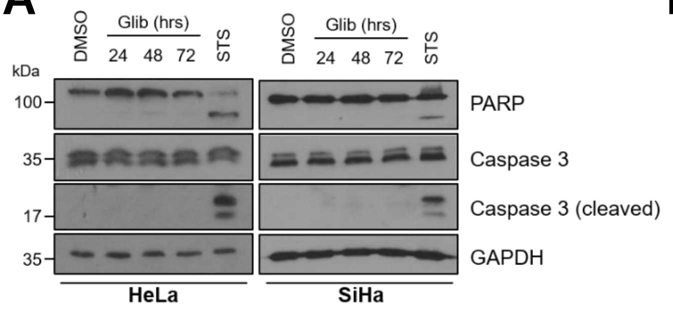

B

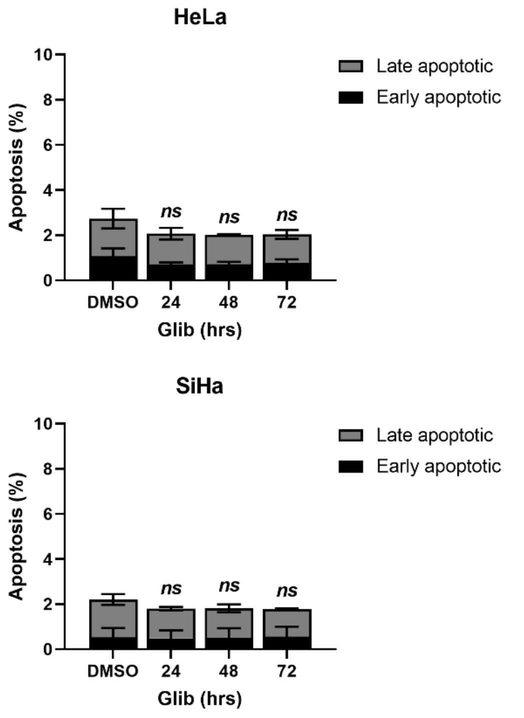

Supplementary Table 1

Transcript	Forward primer (5'-3')	Reverse primer (5'-3')
HPV16 *E6*	CTGCAATGTTTCAGGACCCAC	GTTGTTTGCAGCTCTGTGCAT
HPV16 *E7*	ATTAAATGACAGCTCAGAGGA	GCTTTGTACGCACAACCGAAGC
HPV18 *E6*	TGGCGCGCTTTGAGGA	TGTTCAGTTCCGTGCACAGATC
HPV18 *E7*	GACCTAAGGCAACATTGCA	GCTCGTGACATAGAAGGTC
KCNJ8	CTGGCTGCTCTTCGCTATC	AGAATCAAAACCGTGATGGC
KCNJ11	CCAAGAAAGGCAACTGCAACG	ATGCTTGCTGAAGATGAGGGT
ABCC8	GGTGACCGAATCCCACCATC	CAGGGCAATTAGCAGCTTGG
ABCC9A	CTGGCTTTCTTCAGAATGGT	AAATACCCTCAGAAAAGACTAAAAC
ABCC9B	TGTGATGAAGCGAGGAAATA	TGACACTTCCATTCCTGAGAGA
GFP	ACGTAAACGGCCACAAGTTC	AAGTCGTGCTGCTTCATGTG
CCNA2	TGGAAAGCAAACAGTAAACAGCC	GGGCATCTTCACGCTCTATTT
CCNB1	AAGAGCTTTAAACTTTGGTCTGGG	CTTTGTAAGTCCTTGATTTACCATG
CCND1	CCGCTGGCCATGAACTACCT	ACGAAGGTCTGCGCGTGTT
CCNE1	GCCAGCCTTGGGACAATAATG	CTTGCACGTTGAGTTTGGGT
U6	CTCGCTTCGGCAGCACA	AACGCTTCACGAATTTGCGT
